# Facial and Scalp Swelling in the Pediatric Population With Hemophilia: A Diagnosis Pitfall

**Published:** 2016-04-28

**Authors:** W. Widjaja, S. Aggarwala, F. Ballieux, J. Vandervord

**Affiliations:** The Children's Hospital at Westmead, Westmead, Australia

**Keywords:** hemophilia, facial swelling, coagulopathy, lymphatic malformation, venous malformation

## DESCRIPTION

A 7-month-old boy with unilateral cheek swelling received prompt diagnosis of hemophilia B. He was managed conservatively with an uneventful recovery. In contrast, a 22-month-old boy with scalp swelling had a delay in his diagnosis of hemophilia B. He underwent surgical drainage. Consequently, his hematoma reaccumulated and required blood transfusion.

## QUESTIONS

1. **What are the important red flags in facial or scalp swelling?**
2. **What important differential diagnosis should you consider?**
3. **How should facial and scalp swelling be investigated?**
4. **As a surgeon in training, how should you manage a bleeding diathesis?**

## DISCUSSION

Important red flags to consider are low impact trauma, extension of swelling pass 24 to 48 hours, first incident of trauma, and miscorrelation of clinical history to examination finding in regard to the severity and size of swelling.

Important differential diagnosis to consider should include bleeding diathesis, venous malformation (VM), and lymphatic malformation (LM). Hemophilia is the most common congenital coagulopathy. The incidence of hemophilia B is 1:25000 to 1:30000, also known as Christmas disease.^1^ This is a rare hereditary x-linked recessive bleeding disorder resulting in a deficiency of factor IX. However, 30% of hemophilia B is a result of spontaneous gene mutation of X-chromosome.^2^ The presentation can manifest in a range of clinical spectrum from mild to severe disease, which correlates to the level of factor IX activity. Severe hemophilia B has a factor activity with less than 1% (<0.01 units/mL), which often presents with spontaneous bleeding into joints, muscles, or soft tissue, leading to life-threatening hemorrhages.^1^^,^^3^ Fifty percent to 60% of hemophilia B has moderate to severe disease. Venous malformation can often manifest in the postpartum period after triggers such as trauma or puberty. It continues to develop as the child grows. The hallmarks signs of VM are blue discoloration of the skin, a compressible lump but not pulsatile on palpation and can be painful if thrombosis occurs.^4^ Venous malformations tend to fill on the Valsalva maneuver and exsanguinate on compression, bruits and thrills may not be detected, as lesions are slow-flow. Lymphatic malformations are caused by abnormal development of the lymphatic system. They can have a similar presentation to VM as it often bleeds and is prone to infection preceding trauma.^5^ The diagnosis of LM is based on clinical examination; soft, spongy with tiny raised vesicles overlaying the skin are often associated with cellulitis.^5^^,^^6^

When red flags are raised, initial blood work and ultrasonography provide vital information that would lead to prompt diagnosis and appropriate management. Hemophilia commonly presents with an elevated activated partial thromboplastic time and normal limits of D-dimer and fibrinogen levels. Anemia is associated if there is a significant amount of bleeding. Venous malformation presents with an unremarkable blood cell count. However, in regard to the coagulation profile, D-dimer has been shown to be permanently elevated (>1.8 μg/mL) with accompanying low fibrinogen levels in VM.^7^ This is because VM is associated with spontaneous thrombosis and thrombolysis, a phenomenon known as localized intravascular coagulopathy. The activation of coagulation is likely due to blood stagnation in the enlarged venous channels.^7^ Lymphatic malformation presents with unremarkable blood cell count and coagulation profile. The initial investigation would require an ultrasound examination to demonstrate the multicystic component of LM.

Early consultation with a hematologist is crucial in the initial management of an expanding hematoma in a patient suspicious or known to have a bleeding diathesis. Hematoma in hemophiliacs should be managed conservatively with the replacement of deficient clotting factor. Invasive procedure to drain the hematoma should be avoided as it could lead to life-threatening hemorrhage and unnecessary blood transfusion.

In summary, the case series gives a juxtaposition to illustrate the diagnosis pitfall when faced with common presentations of facial and scalp swelling in the pediatric population. It is important to keep an open mind about possible differential diagnosis and be vigilant about the red flags. Without a family history of bleeding disorder, the diagnosis of bleeding diathesis could be easily overlooked and subsequent surgical management would have been catastrophic and potentially detrimental.

## Figures and Tables

**Figure 1:**
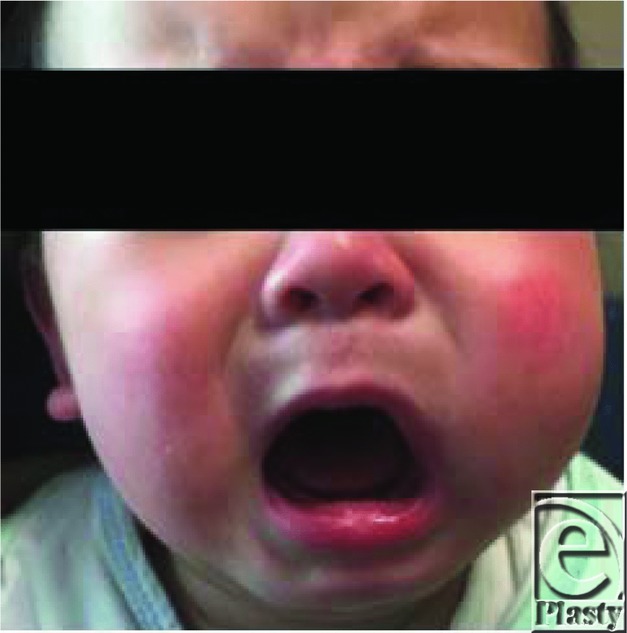
The patient on presentation with unilateral left cheek swelling.

**Figure 2:**
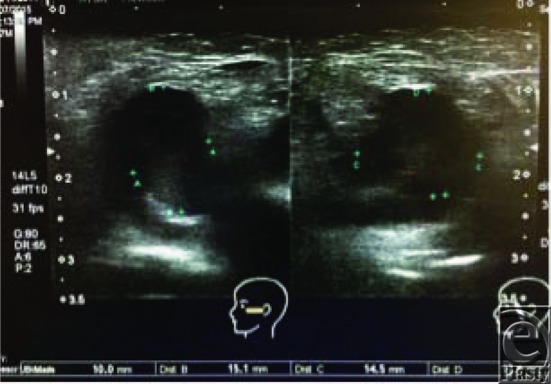
Ultrasound showing heterogeneous mass on corresponding left cheek.

**Figure 3:**
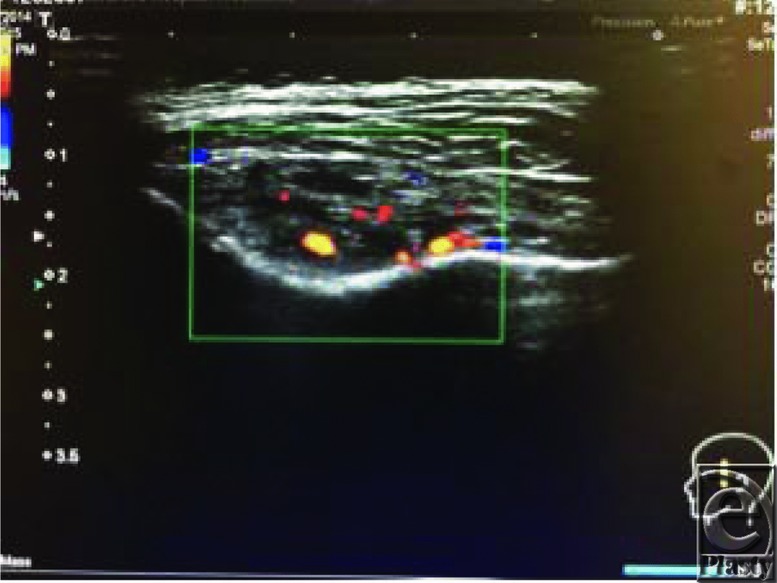
Ultrasound showing heterogeneous mass with no increased vascularity on duplex scanning.
